# PGRMC1 and PGRMC2 in uterine physiology and disease

**DOI:** 10.3389/fnins.2013.00168

**Published:** 2013-09-19

**Authors:** James K. Pru, Nicole C. Clark

**Affiliations:** Department of Animal Sciences, School of Molecular Biosciences, Center for Reproductive Biology, Washington State UniversityPullman, WA, USA

**Keywords:** PGRMC1, PGRMC2, pregnancy, progesterone, uterus

## Abstract

It is clear from studies using progesterone receptor (PGR) mutant mice that not all of the actions of progesterone (P4) are mediated by this receptor. Indeed, many rapid, non-classical P4 actions have been reported throughout the female reproductive tract. Progesterone treatment of *Pgr* null mice results in behavioral changes and in differential regulation of genes in the endometrium. Progesterone receptor membrane component (PGRMC) 1 and PGRMC2 belong to the heme-binding protein family and may serve as P4 receptors. Evidence to support this derives chiefly from *in vitro* culture work using primary or transformed cell lines that lack the classical PGR. Endometrial expression of PGRMC1 in menstrual cycling mammals is most abundant during the proliferative phase of the cycle. Because PGRMC2 expression shows the most consistent cross-species expression, with highest levels during the secretory phase, PGRMC2 may serve as a universal non-classical P4 receptor in the uterus. While the functional importance of PGRMC1/2 in the uterus remains to be fully explored, accumulating evidence suggests that disruption in PGRMC1/2 expression correlates with uterine disease. In this review we will summarize what is known about PGRMC1/2 in uterine physiology and we will provide examples of disrupted expression of these genes in uterine disease states.

## Introduction

The uterus is a primary target of ovarian-derived progesterone (P4), which acts to prepare the endometrium for embryo implantation. In invasively implanting species such as rodents, rabbits, primates and humans, P4 facilitates and maintains the process of endometrial stromal cell decidualization (i.e., terminal differentiation of stromal cells), a critical event that is required for normal pregnancy. In these species, the embryo significantly modifies or, in some instances, completely invades the maternal uterine vasculature in an effort to gain access to oxygen and nutrients. By regulating stromal cell decidualization, P4 contributes to this process and provides balance to ensure that sufficient, but not excessive, invasion of the embryonic trophectoderm occurs. In most mammals, P4 also promotes uterine growth, modulates the maternal immune system locally so the histocompatibly distinct embryo can survive and suppresses myometrial contractions during pregnancy (Graham and Clark, 1997).

Through pharmacologic approaches and mouse mutagenesis studies, many of the actions of P4 are now known to be mediated by the classical P4 receptor (PGR; Lydon et al., 1996; Mulac-Jericevic and Conneely, 2004; Wetendorf and DeMayo, 2012). Indeed, female mice deficient in PGR are sterile (Lydon et al., 1995) and develop endometrial hyperplasia in response to combined estradiol and progesterone treatment likely due to the unopposed actions of estradiol (Mulac-Jericevic et al., 2000). Since its initial description in rodents (Milgrom and Baulieu, 1970; Milgrom et al., 1970) and then later in humans (Rao et al., 1974), PGR has been characterized as a ligand activated transcription factor that functions to regulate gene expression. However, non-genomic actions are also described for PGR where SRC tyrosine kinase activity and subsequent activation of the MAPK pathway has been demonstrated (Migliaccio et al., 1998; Lange, 2004). These and many other studies have unequivocally established PGR as an important mediator of P4 actions in the female.

In accordance with its elementary role in reproduction, faulty P4 responses have been linked to many reproductive diseases that result in subfertility or infertility including fibroids, breast and endometrial cancers, endometriosis, irregular menstrual bleeding, adenomyosis, miscarriage and preterm labor (Szekeres-Bartho et al., 2001; Wu et al., 2006; Burney et al., 2007; Ehn et al., 2007; Ito et al., 2007; Salazar and Calzada, 2007; Boruban et al., 2008). Conceptions that fail due to faulty communication between the mother and embryo remain a major impediment to successful pregnancy, particularly in an IVF setting. As the uterus is a principal target of P4 responses, it is perhaps not surprising that many causes of infertility stem from disrupted P4 actions in the uterus. Epidemiological studies in humans (Christiansen et al., 2005) and livestock (Inskeep and Dailey, 2005), as well as genetic studies in rodents (Conneely et al., 2002; Wang and Dey, 2006), support the notion that failed pregnancy occurs due to faulty uterine function or miscommunication between the embryo and mother during implantation. An estimated 25–60% of conceptions result in pregnancy failure depending upon the mammalian species. In humans, recurrent pregnancy loss occurs at a rate of 1% and is among the most common complications to pregnancy. Importantly, among pregnancies that fail, most occur as pregnancy is being established, long before the placenta develops.

Despite the essential role that PGR plays in female reproduction, not all of the physiological actions of P4 can be explained by activation of PGR. While classical PGR is required for many components of female reproductive physiology and also initiates actions in the male (Luetjens et al., 2006), studies using PGR mutant mice suggest that non-classical P4 signaling mechanisms exist (reviewed in Losel et al., 2003), as outlined in greater detail below. The objective of this perspective article will be to provide examples of non-classical progesterone signaling events *in vivo* and to describe what is currently known about two related non-classical progesterone receptors called progesterone receptor membrane component (PGRMC) 1 and PGRMC2.

## Evidence for non-classical progesterone receptor signaling

Functional studies in *Pgr* null female mice, as well as pharmacological studies using PGR antagonists (e.g., mifepristone) have clearly demonstrated a fundamental role for PGR in female fertility (Conneely et al., 2002). However, PGR does not appear to be the sole receptor mechanism for eliciting P4 actions, as cells that completely lack expression of PGR are still able to respond to P4, as well as to non-metabolizable P4 analogs such as R5020 (Peluso, 2006, 2007; Peluso et al., 2009a,b). Non-classical actions of P4 are historically well-documented in general terms in conjunction with meiotic maturation (Finidori-Lepicard et al., 1981), sexual behavior (Frye et al., 2006) and the acrosome reaction (Foresta et al., 1993; Losel et al., 2004), as well as regulating ion flux in epithelial cells (Head et al., 1999), neurons (Viero et al., 2006), and vascular smooth muscle (Barbagallo et al., 2001). Several laboratories have also shown that P4 modulates immune cell functions in cells completely devoid of classical nuclear PGR (Ehring et al., 1998). Interestingly, these examples of responses to P4 are generally rapid and often do not require gene transcription (i.e., non-genomic actions). Perhaps the most well-documented examples of PGR-independent signaling in cells of the female reproductive system derive from the Peluso lab. Here, studies in the ovary reveal that granulosa cells lacking PGR are resistant to apoptosis in response to various forms of stress in the presence of P4 despite a complete absence of the PGR (Peluso and Pappalardo, 1998; Peluso et al., 2006, 2008, 2009a,b, 2010).

A number of studies have demonstrated non-classical P4 responses in the uterus. For example, the effect of P4 on uterine sensitivity to oxytocin involves direct, but non-classical action of P4 on the uterine oxytocin receptor (Grazzini et al., 1998; Dunlap and Stormshak, 2004; Duras et al., 2005; Bishop and Stormshak, 2006). The details of these studies are discussed in an accompanying review in this edition. DeMayo and colleagues demonstrated that while PGR is important for mediating changes in the expression of many P4-regulated genes in the murine uterus, many other genes are regulated by P4 by a PGR-independent mechanism. Indeed, 44 genes were shown to be differentially regulated in the uteri of *Pgr* null mice in response to P4 treatment (Jeong et al., 2005). Interestingly, these data were derived from mouse U74Av2 microarrays which were spotted with only 6000 known genes along with 6000 ESTs. Collectively, this only constitutes about one third of the entire mouse genome. As such, it is likely that many more genes are regulated by P4 in a PGR-independent manner. In support of this, Matumoto et al. established that P4 up-regulates the Indian Hedgehog (IHH) signaling pathway (Matsumoto et al., 2002). This pathway plays an integral role in coordinating uterine epithelial-mesenchymal interactions during embryo implantation. In their study, ovariectomy of wild type mice followed 1 week later with a single injection of P4 resulted in transcriptional up-regulation of *Ihh* mRNA. *Ihh* mRNA was shown by *in situ* hybridization to be up-regulated within 6 h. The same treatment was given to *Pgr* null mice, and surprisingly *Ihh* was also up-regulated within 6 h despite a complete absence of PGR from the uterus. Similar results were found for other members of the IHH pathway like Patched-1 (*Ptc1*), as well as the downstream IHH target homeobox gene A10 (*Hoxa10*). The important conclusion from these studies is that the transcriptional effects of P4 on *Ihh*, *Ptc*, and *Hoxa10* in the uterus are mediated in part by a PGR-independent mechanism.

These data clearly illustrate that P4 actions are mediated by multiple signaling pathways that involve both PGR and other, as yet, undefined pathways. Two families of non-classical membrane receptors have been identified and these include the Progestin and AdipoQ receptor (PAQR) and Progesterone Receptor Membrane Component (PGRMC) families (Tang et al., 2005; Cahill, 2007; Peluso, 2007; Thomas, 2008; Thomas and Pang, 2012). Members of the PAQR family with purported P4 binding activity belong to the G protein-coupled receptor superfamily and include membrane progestin receptors α, β, and γ, (also called PAQR VII, VIII, and V, respectively). Each gene has been cloned and partially characterized in mammals (Zhu et al., 2003). While these receptors have been shown to have biological actions *in vitro* (Karteris et al., 2006), others have challenged the validity of the mPRs as *bona fide* progestin receptors (Krietsch et al., 2006). In addition, two distinct proteins with progestin binding activity have been described and these are referred to as PGRMC1 and PGRMC2. These genes were originally cloned as hem-1 domain protein or HPR6.6 and Dg6, respectively (Falkenstein et al., 1996; Meyer et al., 1996; Gerdes et al., 1998). The function of each of these genes has been outlined in two recent review articles (Cahill, 2007; Wendler and Wehling, 2013).

## PGRMC1 and PGRMC2 expression and function in the uterus

PGRMC1 and PGRMC2 are highly expressed in female reproductive tissues of the mouse (Zhang et al., 2008), rat (Peluso et al., 2006; Intlekofer and Petersen, 2011; Lodde and Peluso, 2011), monkey (Keator et al., 2012), cow (Luciano et al., 2010, 2011; Slonina et al., 2012), and human (Engmann et al., 2006; Zhang et al., 2008; Peluso et al., 2009a,b).

### PGRMC1

The first report of PGRMC1 expression in the uterus derives from a microarray study in which *Pgrmc1* mRNA was found to be down-regulated from the proliferative (i.e., epithelial cell cycle progression, mucosal edema and angiogenesis) to the secretory (i.e., mitosis blockade, cellular differentiation and mucosal secretion) phase of the human menstrual cycle (Kao et al., 2002). This was later confirmed with microarray data in the primate (Ace and Okulicz, 2004) and a proteomics-based study in women (Chen et al., 2009). Beyond this, very little is known about PGRMC1 expression in the human endometrium. Keator et al. recently demonstrated that PGRMC1 mRNA and protein are highly expressed in the proliferative phase in an artificial menstrual cycle model in monkeys, but then becomes down-regulated to the point of not being detected during the late secretory phase (Keator et al., 2012). Expression of PGRMC1 during the proliferative phase was most evident in the stroma, glandular epithelium and luminal epithelium of the inner-most aspect of the endometrium (i.e., functionalis). Given that PGRMC1 is most abundant during the proliferative phase of the human and monkey menstrual cycle, it may serve a role in regulating the cell cycle. PGRMC1 is also expressed and highly regulated in the human decidua at the maternal:fetal interface, as well as in the embryonic/fetal trophectoderm (Zhang et al., 2008). In each of these cases, the subcellular localization of PGRMC1 was most abundant in the peri-nuclear region.

Interestingly, despite the dynamic regulation of PGRMC1 in the primate and human, PGRMC1 expression does not change during the estrous cycle in the cow (Luciano et al., 2011; Kowalik et al., 2013). However, in the mouse PGRMC1 expression is highest when female mice are exposed to P4 during either the estrous cycle or following ovariectomy and subsequent P4 supplementation (Zhang et al., 2008). Within the endometrium, PGRMC1 expression is highly regulated and dependent on the stage of the estrous cycle, pregnancy status and steroid hormone supplementation following ovariectomy (Zhang et al., 2008). Specifically, the PGRMC1 expression pattern changed from mainly the glandular and luminal epithelium during protestrus to then also include stromal cells during metestrus. As seen with granulosa-to-luteal cell differentiation in the ovary, the cellular localization of PGRMC1 changes during stromal cell decidualization of early pregnancy in which the protein transitions from the plasma membrane of undifferentiated endometrial stromal cells to the nuclei of stromal cells undergoing differentiation. Upon terminal stromal cell differentiation (i.e., decidualizatioin), PGRMC1 becomes localized throughout the cell, particularly in the peri-nulcear space. Because of the observed nuclear localization, we speculate that PGRMC1 regulates expression of a unique set of genes distinct from those regulated by the classical PGR. In support of this, PGRMC1 was recently shown to regulate expression of genes associated with apoptosis (Peluso et al., 2010), as well as activity of the TCF/LEF transcriptional unit in granulosa cells (Peluso et al., 2012). Alternatively, PGRMC1 may participate in cell cycle regulation given that nuclear localization occurs at a time when proliferative stromal cells transition to terminally differentiated decidual cells. A role for PGRMC1 in regulating cell cycle progression has been proposed in granulosa cells (Lodde and Peluso, 2011).

### PGRMC2

Considerably less information is available on PGRMC2 expression and function. Recently, Albrecht et al. (2012) demonstrated in SKOV-3 cancer cells that PGRMC2 may function to inhibit cell migration (Wendler and Wehling, 2013). Within the uterus, *Pgrmc2* mRNA is up-regulated by P4 in both mice and monkeys. In the mouse, uterine PGRMC2 expression is elevated during metestrus, as well as in response to P4 treatment following ovariectomy (Zhang et al., 2008). Similarly, PGRMC2 expression increases substantially during the secretory phase of an artificial menstrual cycle in macaques (Keator et al., 2012). In this model, PGRMC2 mRNA and protein localize strongly to the functionalis layer, particularly the glandular and luminal epithelial compartments. This corresponds to a time when PGRMC1 and the classical PGR are absent. As such, while several studies have demonstrated that the stromal compartment indirectly mediates the actions of P4 on the epithelium via paracrine signaling, P4 may also directly signal in epithelial tissue at the time of embryo implantation via PGRMC2-mediated signaling. A significant increase in PGRMC2 was also observed in the human choriodecidua of term and pre-term pregnancies (Shankar et al., 2010).

The expression studies reveal two important points regarding PGRMCs. First, uterine expression of PGRMC1 and PGRMC2 is regulated by endocrine factors. This is evident from the dynamic patterns of PGRMC1 and PGRMC2 expression under physiological conditions and in response to steroid hormones. Second, much work is clearly needed, particularly in humans, to fully characterize the pattern of PGRMC1 and PGRMC2 expression in the mammalian uterus and to establish a functional role for these genes in female reproduction. The most recent overview of general PGRMC1 and PGRMC2 functions is outlined in a recent review (Wendler and Wehling, 2013).

## Relationships between disrupted PGRMC1 and PGRMC2 expression and the development of disease states

Several recent studies have suggested that PGRMC1 and PGRMC2 are important for maintaining normal reproductive functions. For instance, *Pgrmc1* levels are reduced in peripheral blood cells in women with polycystic ovarian syndrome (Schuster et al., 2010) and in some women with premature ovarian failure (Mansouri et al., 2008; Schuster et al., 2010). In contrast PGRMC1 over-expression is associated with impaired follicular development in women induced to undergo ovulation as part of their infertility treatment (Elassar et al., 2012). Antral follicle development is impaired in mice in which the *Pgrmc1* gene has been conditionally knocked out (cKO) of granulosa cells (Pru and Peluso unpublished).

**Figure 1:**
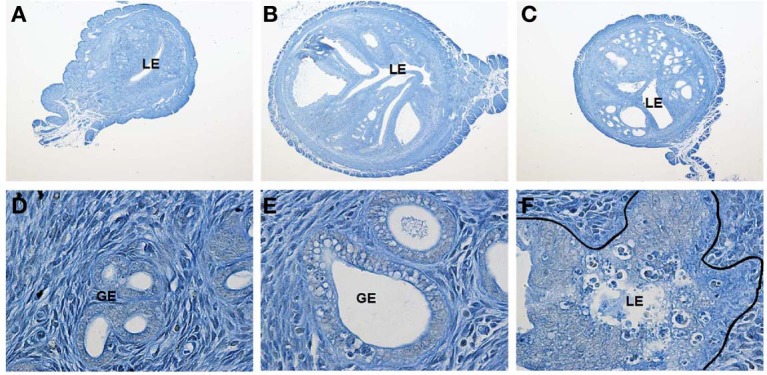
**Female *Amhr2^cre/+^; Pgrmc1^fl/fl^* conditional knockout (cKO) mice develop uterine cystic hyperplasia**. Shown are uterine cross-sections of control (*Amhr2^+/+^;Pgrmc1^fl/fl^*, **A**) and *Pgrmc1* cKO (*Amhr2^cre/+^;Pgrmc1^fl/fl^*; **B** and **C**) female mice at 6 months of age (40X). Extensive cystic hyperplasia is evident in glandular tissue in sections from cKO mice. Higher magnification images (600X) reveal normal glandular tissue in control **(D)** sections, with development of vacuoles in glandular epithelium in sections from cKO mice **(E)**. Epithelial cells with pyknotic nuclei consistent with apoptosis, disruption of the transitional zone between epithelial and stromal tissues and infiltration of immune cells are also evident in sections obtained from cKO female mice **(F)**.

As noted above, PGRMC2 expression is increased by P4 in the luminal and glandular epithelia of the secretory endometrium in the macaque (Keator et al., 2012). In contrast, the level of PGRMC2 is reduced and its cellular localization disrupted in a primate model of endometriosis (Keator et al., 2012). Importantly, this phenotype is recapitulated in women with endometriosis (Bunch et al., 2013). This suggests that aberrant PGRMC2 expression may contribute to P4 refractoriness commonly found in endometriosis. Within the ovary, *Pgrmc2* expression is elevated in granulosa cells of young women with diminished ovarian reserve. Previously unpublished data from our lab reveal that PGRMC1 deficiency in the murine endometrium results in development of cystic glandular hyperplasia by as early as 3 months of age (Figure 1). By 6 months of age, greater than 80% of the female mice exhibit the phenotype. While control female mice display with expectedly normal endometrium, female mice in which *Pgrmc1* was conditionally deleted from mesenchymal tissue (i.e., stroma and myometrium) of the uterus using the *anti-Mullerian hormone type II receptor-cre recombinase* (*Amhr2-cre*) transgenic mouse develop hyperplastic and enlarged glands. The glandular hyperplasia is accompanied by the presence of many epithelial cells with pyknotic nuclei indicative of apoptotis, infiltration of immune cells, heavily vacuolated epithelial cells, and disruption of the transitional zone between the epithelium and underlying stromal tissue. Interestingly, many cross sections obtained from *Pgrmc1* cKO mice harbor multiple glandular structures that are larger than even the luminal space. This accounts for the approximate 2-fold increase in cross-sectional volume compared with control sections. This is a complex phenotype given that epithelial disorder occurs when *Pgrmc1* is deleted from the surrounding mesenchymal tissue. Clearly more studies are needed, but we speculate that paracrine signaling between the stromal and epithelial compartments is disrupted in *Pgrmc1* cKO mice. In the very least, it is clear that stromal PGRMC1 is necessary for cross-talk between the mesenchymal and epithelial compartments. These cumulative findings clearly demonstrate that PGRMC1 and PGRMC2 play important and clinically relevant roles in regulating female reproductive functions and in the manifestation of disease states in female reproductive tissues when normal expression is disrupted.

### Conflict of interest statement

The authors declare that the research was conducted in the absence of any commercial or financial relationships that could be construed as a potential conflict of interest.
